# Clinical analysis of seven cases of primary hepatic neuroendocrine neoplasms

**DOI:** 10.3389/fonc.2026.1729932

**Published:** 2026-03-25

**Authors:** Mengting Zhang, Lunan Wu, Lei Wu, Bingsong Yan

**Affiliations:** 1 Department of Hepatobiliary Surgery, The Affiliated Hospital of Qingdao University, Qingdao, China; 2 Department of Thoracic Surgery, Women and Children’s Hospital Affiliated to Qingdao University, Qingdao, China; 3 Department of General Surgery, Peking University People’s Hospital, Qingdao Hospital, Qingdao, China; 4 Department of Hepatobiliary Surgery, Women and Children’s Hospital Affiliated to Qingdao University, Qingdao, China

**Keywords:** diagnosis, pathology, primary hepatic neuroendocrine neoplasm, prognosis, surgical treatment

## Abstract

**Objective:**

To summarize the clinical manifestations, imaging features, laboratory findings, pathological characteristics, treatment modalities, and prognosis of primary hepatic neuroendocrine neoplasms (PHNEN).

**Methods:**

A retrospective analysis was conducted on the clinical data of 7 patients with PHNEN admitted to The Affiliated Hospital of Qingdao University between June 2015 and July 2023. Data collected included clinical manifestations, imaging features, and laboratory results.

**Results:**

The cohort consisted of 4 males and 3 females, with a median age of 54 (53, 64) years. Six patients were asymptomatic, with lesions discovered incidentally during physical examinations, one patient presented with an upper abdominal mass. Six patients had solitary tumors (3 in the left lobe, 3 in the right lobe), and one patient had multiple tumors involving both lobes. Pathologically, there were 5 cases of neuroendocrine tumors (NET), including 4 G2 grade and 1 G1 grade, and 2 cases of Small cell neuroendocrine carcinoma (SCNEC). Arterial phase enhancement was observed in 3/3 patients undergoing contrast-enhanced CT and in 3/4 patients undergoing contrast-enhanced MRI. Immunohistochemical staining showed positivity rates of 7/7 for Syn, 5/5 for CgA, and 5/5 for CD56. Six patients underwent radical surgical resection, while one patient only underwent intraoperative biopsy due to excessively large tumor size. The median follow-up time was 11 (3, 102) months. Among the six patients who underwent radical resection, four showed no recurrence, one recurred at 8 months postoperatively, and one recurred at 96 months postoperatively. These two patients received no further treatment after recurrence and died 2 months and 6 months later, respectively. The patient who only underwent biopsy died 3 months postoperatively.

**Conclusion:**

PHNEN lacks specific clinical manifestations, imaging findings, and laboratory test results. Definitive diagnosis relies on pathological examination. Radical surgical resection is an effective treatment.

## 1 Introduction

Primary hepatic neuroendocrine neoplasm (PHNEN) is a highly heterogeneous tumor originating from neuroendocrine cells (1). Most hepatic neuroendocrine tumors are metastases from primaries in the gastrointestinal tract, pancreas, or lungs, making PHNEN extremely rare. Approximately 150 cases have been reported worldwide (2), accounting for about 0.4% of all neuroendocrine tumors (3). Previous reports have mostly been case studies. This paper retrospectively analyzes the data of 7 PHNEN patients, summarizing their clinical features, laboratory tests, imaging characteristics, and pathological findings to provide references for the clinical diagnosis and treatment of PHNEN.

## 2 Materials and methods

### 2.1 General information

A retrospective analysis was performed on the clinical data of 7 patients with PHNEN admitted to the Department of Hepatobiliary Surgery, The Affiliated Hospital of Qingdao University, between June 2015 and July 2023. Inclusion criteria: (1) Pathological diagnosis of PHNEN; (2) Exclusion of other liver tumors (e.g., primary liver cancer, intrahepatic cholangiocarcinoma, hepatic hemangioma); (3) No history of malignant tumors; (4) Complete clinical data available. Exclusion criteria: (1) Discovery of an extrahepatic primary lesion during preoperative diagnosis or postoperative follow-up; (2) Concurrent malignancy in other sites. The cohort included 4 males and 3 females, with a median age of 54 (53, 64) years. This study was approved by the Ethics Committee of The Affiliated Hospital of Qingdao University (Approval No: QYFY-WZLL-30581), and informed consent was obtained from all patients.

### 2.2 Exclusion of extrahepatic primary tumor

All seven patients underwent routine preoperative chest, abdominal, and pelvic computed tomography (CT), with no evidence of extrahepatic primary malignancy. Gastroscopy and colonoscopy were not routinely performed preoperatively; only four patients received these examinations, which revealed no extrahepatic primary tumor. Owing to limited equipment availability at the time, Somatostatin receptor imaging was not performed in any of the patients and only one patient underwent postoperative positron emission tomography/computed tomography (PET/CT). No abnormal tracer uptake was identified in the extrahepatic digestive and respiratory systems, thereby excluding an extrahepatic primary malignancy.

### 2.3 Observation indicators

Clinical data, including patient complaints, past medical history, tumor diameter, number of tumors, metastasis sites, and immunohistochemistry results were collected. Follow-up was conducted by reviewing medical records, outpatient data, and telephone interviews until June 2024, or until patient loss to follow-up or death.

### 2.4 Tumor grading standard

According to the 2019 WHO classification of digestive system tumors, PHNENs were classified into neuroendocrine tumors (NET G1, G2, G3), Small cell neuroendocrine carcinoma (SCNEC), Large cell neuroendocrine carcinoma (LCNEC), and mixed neuroendocrine-non-neuroendocrine neoplasms (MiNEN) (4).

### 2.5 Statistical analysis

SPSS 22.0 software and GraphPadPrism were used for statistical analysis. Normally distributed measurement data were expressed as mean ± standard deviation, while non-normally distributed data were expressed as median (Q1, Q3). Survival curves were generated by the Kaplan-Meier method.

## 3 Results

### 3.1 Clinical manifestations

The cohort consisted of 4 males and 3 females, with a median age of 54 (53, 64) years. Six patients were incidentally found to have liver space-occupying lesions during physical examinations, and one patient presented with an upper abdominal mass. Past medical history included hypertension in one patient, diabetes in one patient, both hypertension and diabetes in one patient, and hepatitis B in one patient. The median tumor diameter was 6 (2, 9) cm. Six cases were solitary tumors (3 in the left lobe, 3 in the right lobe), and one case had multiple tumors involving both lobes. This patient with multiple tumors was found to have gallbladder metastasis during surgery, while the other six had no extrahepatic metastases (**Table 1**).

**Table 1:** Clinical data of 7 patients with primary hepatic neuroendocrine neoplasms.

Gender	Age (years)	Clinical manifestation	Medical history	Tumor diameter (cm)	Tumor location	Number of tumors	Metastasis site
Female	53	Physical exam finding	None	2.0	Right lobe	Single	None
Female	56	Physical exam finding	None	6.0	Bilateral	Multiple	Gallbladder
Male	53	Physical exam finding	Hypertension	3.0	Left lobe	Single	None
Male	64	Physical exam finding	None	23.0	Left lobe	Single	None
Male	54	Physical exam finding	Hepatitis B	8.0	Left lobe	Single	None
Male	52	Physical exam finding	Diabetes	1.5	Right lobe	Single	None
Female	67	Upper abdominal mass	Hypertension, Diabetes	9.0	Right lobe	Single	None

### 3.2 Imaging examinations

#### 3.2.1 Ultrasonography

Four patients underwent ultrasonography. Three showed hypoechoic nodules in the liver with relatively regular morphology and clear boundaries, two of which had irregular light spots; one presented as a cystic-solid mass, with the cystic part showing multiple septations, poor sound transmission, filled with dense punctate echoes, and the solid part showing punctate and linear blood flow signals (**Figure 1**).

**aFigure 1:**
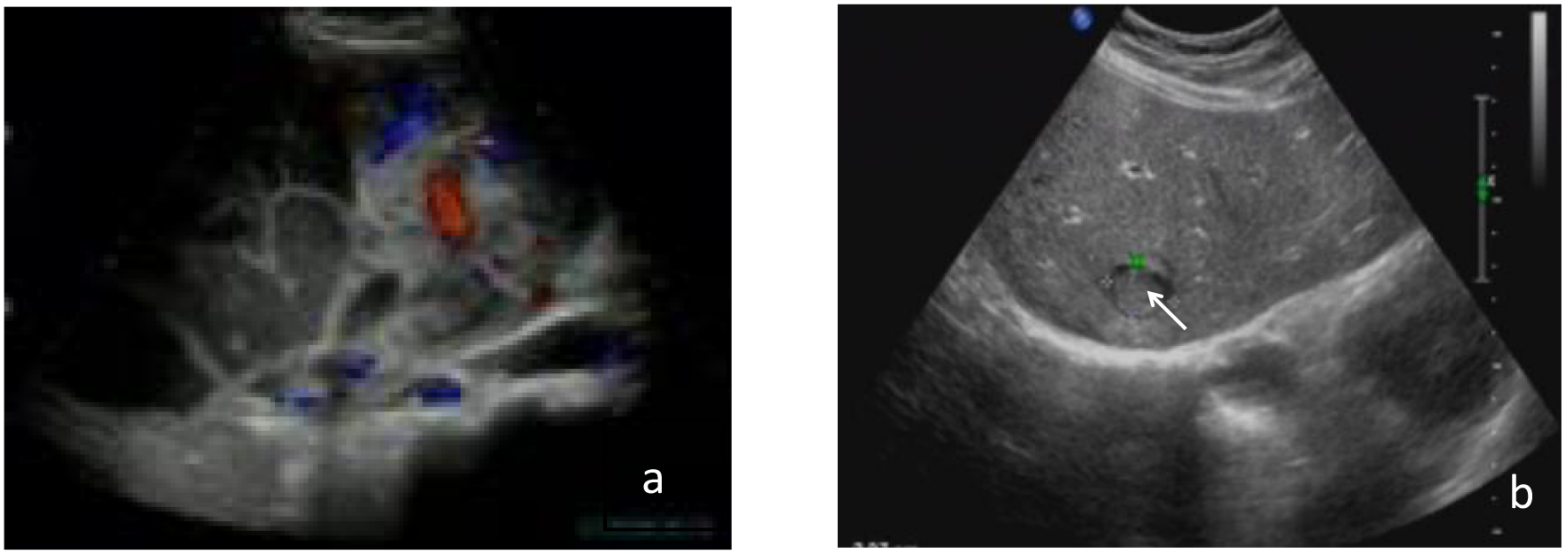
Ultrasonography of PHNEN. **(a)** Cystic-solid mass with internal septations, poor sound transmission, dense punctate echoes, and blood flow signals in the solid part; **(b)** Hypoechoic nodule.

#### 3.2.2 CT examination

**Figure 2:**
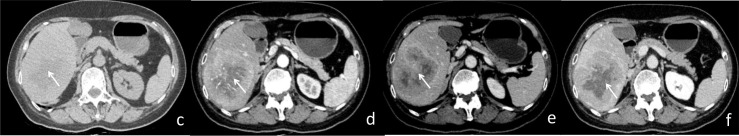
CT examination of PHNEN. **(c)** Plain CT showing poorly defined patchy low-density shadow; **(d)** Arterial phase significant enhancement; **(e)** Venous phase reduced enhancement; **(f)** Delayed phase reduced enhancement.

All patients underwent a plain CT scan showing a poorly defined, patchy, low-density shadow. Three patients underwent abdominal contrast-enhanced CT, all showing heterogeneous density masses with significant enhancement in the arterial phase, and reduced enhancement in the venous and delayed phases (**Figure 2**).

#### 3.2.3 MRI examination

**Figure 3:**
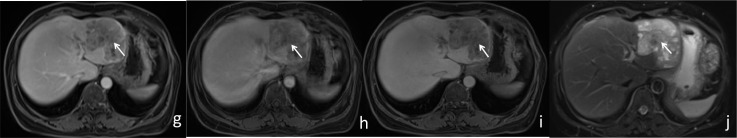
MRI examination of PHNEN. **(g)** Arterial phase obvious heterogeneous enhancement; **(h)** Portal venous phase reduced enhancement; **(i)** Equilibrium phase reduced enhancement; **(j)** T2WI mixed high signal.

Four patients underwent abdominal contrast-enhanced MRI. Three showed obvious heterogeneous enhancement in the arterial phase, with reduced enhancement in the portal venous and equilibrium phases and T2WI mixed high signal (**Figure 3**). One case showed only mild enhancement in the portal venous phase and no enhancement in the arterial phase.

### 3.3 Laboratory tests

All 7 patients underwent tumor marker and liver function tests. Two patients had elevated CA-199, one had elevated CA-125; tumor marker results were normal in the remaining patients. One patient had abnormally elevated ALT, one patient had abnormally elevated ALT combined with slightly low albumin; liver function tests were normal in the remaining patients (**Table 2**).

**Table 2:** Tumor markers and liver function results of 7 PHNEN patients.

Case	Diagnosis	Tumor marker				Liver function			
		AFP (ng/mL)	CEA (ng/mL)	CA199 (U/mL)	CA125 (U/mL)	Albumin (g/L)	Total bilirubin (μmol/L)	ALT(U/L)	AST (U/L)
1	NET,G2	2.88	0.23	9.67	13.18	43.6	13.9	29	25
2	SCNEC	3.52	2.14	17.5	15.11	43.7	16.4	27	24
3	NET,G1	0.83	2.38	46.17	9.26	38.3	19.3	172	40
4	NET,G2	2.77	0.73	10.20	81.96	40.6	33.4	49	41
5	SCNEC	4.12	2.35	40.76	7.60	48.8	17.9	104	46
6	NET,G2	1.29	1.00	6.33	6.60	49.7	17.0	39	31
7	NET,G2	1.46	3.83	28.75	9.70	41.5	9.8	29	38

NET, neuroendocrine tumor; SCNEC, small cell neuroendocrine carcinoma.

### 3.4 Treatment methods and pathological results

Six patients underwent radical surgical resection, and one patient only underwent intraoperative biopsy due to excessively large tumor size. Pathological examination confirmed 5 cases of NET (4 G2 grade, 1 G1 grade) and 2 cases of SCNEC. Immunohistochemical staining showed Syn positivity in 7/7 cases, CgA positivity in 5/5 cases, and CD56 positivity in 5/5 cases (**Table 3**).

**Table 3:** Treatment methods, pathological results, and prognosis of 7 PHNEN patients.

Case	Diagnosis	Treatment methods and pathological results					Prognosis		
		Treatment	ki-67(%)	Syn	CgA	CD56	Recurrence time and treatment	Follow-up time (months)	Status
1	NET,G2	Surgery	6	+	NT	NT	No recurrence	108	Alive
2	SCNEC	Surgery	60	+	+	NT	8 months, No treatment	10	Deceased
3	NET,G1	Surgery	1	+	+	+	No recurrence	11	Alive
4	NET,G2	Biopsy only	8	+	+	+	None	3	Deceased
5	SCNEC	Surgery	60	+	+	+	96 months, No treatment	102	Deceased
6	NET,G2	Surgery	3	+	+	+	No recurrence	12	Alive
7	NET,G2	Surgery	20	+	NT	+	No recurrence	3	Alive

NET, neuroendocrine tumor; SCNEC, small cell neuroendocrine carcinoma; Ki-67, cell proliferation nuclear antigen; Syn, synaptophysin; CgA, chromogranin A; CD56, neural cell adhesion molecule; NTm, Not tested.

### 3.5 Prognosis

**Figure 4:**
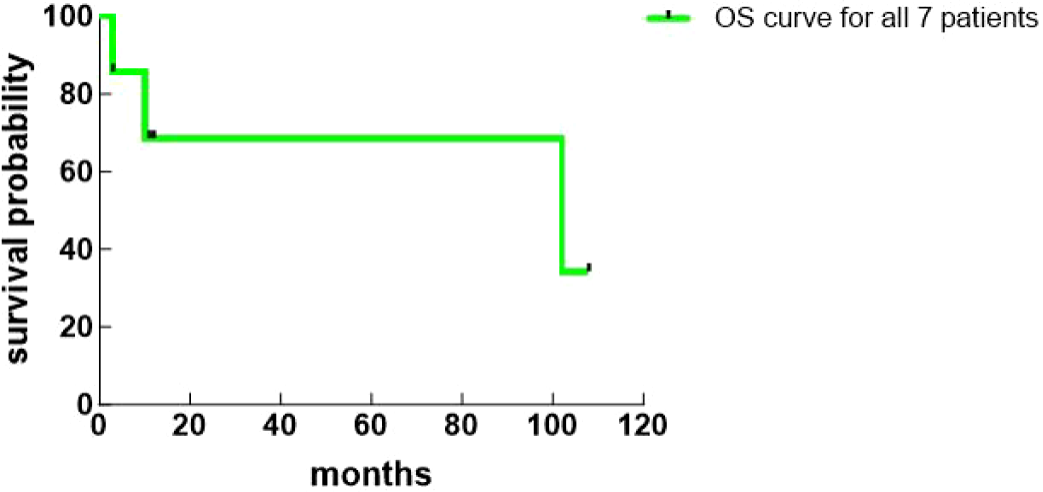
OS curve for all 7 patients (Kaplan-Meier curves are provided for descriptive visualization only due to the small sample size).

**Figure 5:**
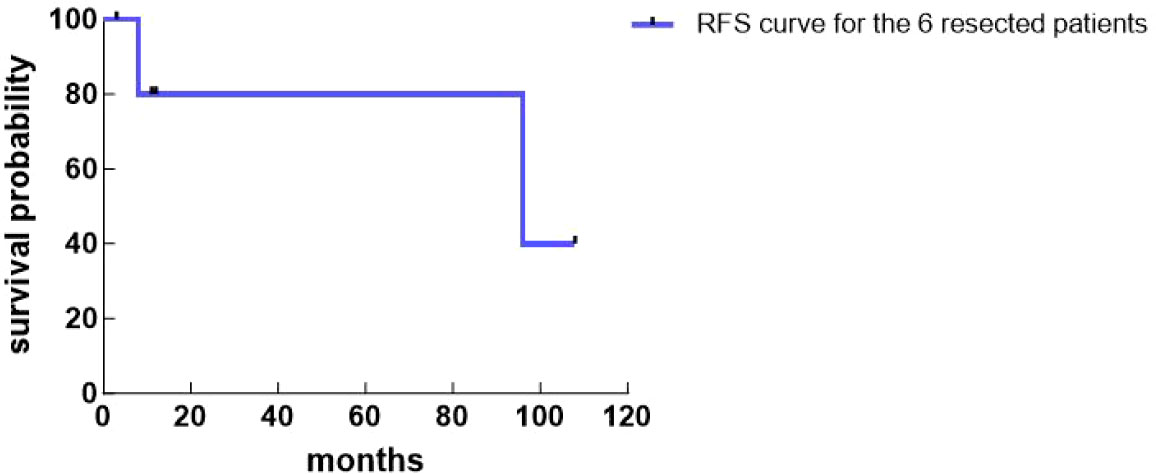
RFS curve for the 6 resected patients (Kaplan-Meier curves are provided for descriptive visualization only due to the small sample size).

The median follow-up time was 11 (3, 102) months. Among the 6 patients who underwent radical resection, 4 showed no recurrence, 1 recurred at 8 months postoperatively, and 1 recurred at 96 months postoperatively. These two patients received no further treatment after recurrence and died 2 months and 6 months later, respectively. The patient who only underwent biopsy died 3 months postoperatively (**Table 3**). The Kaplan-Meier curves were plotted based on the survival of all patients (**Figures 4**, **5**).

## Discussion

PHNEN primarily occurs in middle-aged patients with no significant gender difference. Most patients are asymptomatic and are discovered during physical examinations. A small number of patients may experience abdominal pain or distension. Most PHNENs do not produce hormones and exhibit an indolent clinical course; only about 5% of patients present with carcinoid syndrome (e.g., asthma, diarrhea, facial flushing) (5). In this group, most patients were asymptomatic, 6 were discovered incidentally during physical exams, only 1 sought medical attention due to an abdominal mass, and none presented with carcinoid syndrome.

In this study, one patient with gallbladder involvement was enrolled, who needed to be differentiated from primary gallbladder neuroendocrine carcinoma with liver metastasis. The detailed evidence is as follows:

Pathological evidence: Re-evaluation of pathological sections from the liver-gallbladder junction showed an intact structure of the gallbladder mucosal epithelium, with no dysplasia or carcinoma *in situ*. The main tumor mass was located within the hepatic parenchyma and invaded the gallbladder serosa and outer muscular layer in a pushing pattern, while the gallbladder mucosa remained intact. This growth pattern was more consistent with direct invasion of the gallbladder by a primary hepatic tumor, rather than a malignant tumor originating from the gallbladder mucosa.
Immunohistochemical features: Additional immunohistochemical staining was performed in this case. The results showed diffuse strong positivity for CK7. Combined with positive neuroendocrine markers, the immunophenotype was more in favor of primary hepatic neuroendocrine carcinoma, rather than primary gallbladder neuroendocrine carcinoma.
Radiological review: Imaging examinations revealed a large, dominant hepatic lesion with significant mass effect. The thickened gallbladder wall was closely connected to the hepatic lesion, showing external compression and invasive changes, rather than an independent polypoid or mucosal-origin lesion within the gallbladder lumen. Based on the above pathological, immunophenotypic and radiological evidence, we believe that a diagnosis of primary hepatic neuroendocrine neoplasm with gallbladder invasion is more reasonable for this case.

### 3.6 Laboratory findings

Serum tumor markers and liver function tests are mostly within normal ranges in PHNEN and hold little diagnostic value (6). In this study, AFP and CEA results were normal in all 7 patients, with only one case showing a slight elevation of CA125 and two cases showing a slight elevation of CA199. Previous studies have shown that plasma CgA level has high sensitivity (70%) and specificity (93%) for diagnosing neuroendocrine tumors (7), but its value in definitively diagnosing NENs is limited. Furthermore, some non-NENs, such as prostate cancer and neuroblastoma, can also express CgA, leading to false positives (8). Additionally, the level of 5-hydroxyindoleacetic acid (5-HIAA) in 24-hour urine is significant for diagnosing PHNEN, with a specificity of 90% and sensitivity of 73% (9). In this study, since PHNEN was not considered preoperatively, none of the 7 patients underwent plasma CgA or 24-hour urine 5-HIAA testing.

### 3.7 Imaging characteristics

Ultrasonography often shows slightly hyperechoic masses or mixed echogenicity masses predominantly hyperechoic, with irregular light spots, multiple linear strong echo septa, and rich internal blood flow signals (10). In this study, two cases showed internal irregular light spots, and one case presented as a cystic-solid mass with internal multiple septa, consistent with previous reports. The presence of low echogenicity nodules in this study may be related to the small sample size. On CT, the lesion appears as round or oval low-density shadows within the liver, either solitary or multiple. Solitary lesions are more commonly found in the right lobe, with blurred boundaries and heterogeneous internal density, which may be accompanied by liquefactive necrosis. Contrast-enhanced scans show varying degrees of enhancement at the tumor edge and septa in the arterial phase, no enhancement in the central necrotic area, and persistent but reduced enhancement in the venous phase. MRI often shows mixed high signal on T2WI and mixed low signal on T1WI, with central liquefaction necrosis areas showing long T1 and T2 signals. It has been reported that characteristic imaging findings of PHNEN include: on contrast-enhanced MRI, obvious enhancement at the tumor edge and septa, no enhancement in the necrotic area, and the mass presenting multilocular changes (11).

Pathological examination is the gold standard for diagnosing PHNEN. Chromogranin A (CgA) and synaptophysin (Syn) are the two most commonly used immunohistochemical markers for detecting neuroendocrine tumors in pathological laboratories (12). CD56 is located on the membrane surface of central and peripheral nerve cells and fibers. Some articles report that CD56 positivity in primary hepatic neuroendocrine tumors also holds certain diagnostic significance and can be combined with CgA and Syn to further improve diagnostic accuracy (13). In this group, the immunohistochemical positivity rates were 100% for CgA (5/5), 100% for Syn (7/7), and 100% for CD56 (5/5), consistent with previous research findings.

Treatment methods for PHNEN are diverse, primarily including surgical resection, transarterial embolization (TAE), transarterial chemoembolization (TACE), somatostatin analog (SSA) therapy, peptide receptor radionuclide therapy (PRRT), liver transplantation, and targeted drug therapy. Among these, surgical resection is the preferred treatment. A retrospective single-center study involving 22 PHNEN patients who underwent surgical resection (14) showed 1-year, 3-year, and 5-year recurrence-free survival rates of 86.4%, 63.6%, and 52.9%, respectively. In this study, 5 patients had follow-up times reaching 1 year, 1 experienced tumor recurrence, resulting in a 1-year recurrence-free survival rate of 80%, consistent with previous studies. Due to the low incidence of PHNEN and limited related clinical studies, no consensus has been reached on surgical procedures. Scholars such as Frilling (15) suggest that achieving R0 resection(ensuring negative margins) combined with regional lymph node dissection is the optimal strategy for surgical treatment of PHNEN. If the tumor is too large or invades vital organs or blood vessels, making radical resection impossible, palliative surgery can still alleviate clinical symptoms and improve the patient’s quality of life (16). For patients with inoperable NENs, peptide receptor radionuclide therapy (PRRT) can serve as a neoadjuvant treatment, reducing tumor volume and converting some initially unresectable lesions into resectable ones (17). Furthermore, PHNENs are hypervascular tumors sensitive to ischemia, making TAE and TACE common treatment methods. TAE induces tumor ischemia and necrosis by embolizing the tumor’s blood supply vessels; TACE involves infusing chemotherapy drugs along with embolization to directly kill tumor cells. A retrospective analysis by Pericleous (18) et al. of 50 patients with neuroendocrine tumors confirmed the significant efficacy of TAE/TACE in controlling symptoms and tumor growth. Somatostatin analogs (SSA) bind to somatostatin receptors on the tumor cell surface, not only reducing hormone levels in the blood and alleviating clinical symptoms but also inhibiting tumor progression (19). A randomized controlled double-blind trial indicated that SSA can prolong the survival of patients with neuroendocrine tumors (20). Additionally, liver transplantation can also be a treatment option for PHNEN. In recent years, sorafenib, sunitinib, and bevacizumab have shown some benefit in treating neuroendocrine tumor patients, but data proving their efficacy specifically for PHNEN patients is lacking.

### 3.8 Considerations for systemic/adjuvant therapy and management of recurrence

The European Society for Medical Oncology (ESMO) guidelines recommend cisplatin plus etoposide (EP) or carboplatin plus etoposide (EC) as first-line chemotherapy regimens for advanced neuroendocrine carcinoma (NEC) (21). Li (22) compared the efficacy and prognostic factors of chemotherapy versus transcatheter arterial chemoembolization (TACE) in the treatment of advanced primary hepatic neuroendocrine carcinoma. The results showed that the median overall survival (OS) was 14.8 months in the EP/EC chemotherapy group, which was significantly better than 12.2 months in the TACE group (P = 0.040); the median progression-free survival (PFS) was 4.4 months in the EP/EC group, also significantly higher than 2.7 months in the TACE group (P = 0.005). These findings suggest that the EP/EC regimen may be a preferable therapeutic option for patients with advanced primary hepatic neuroendocrine carcinoma (PHNEC). In the present study, 2 patients with NEC did not receive adjuvant chemotherapy postoperatively. The main reason was that despite repeated communication and recommendations for adjuvant chemotherapy after surgery, the patients and their families showed obvious resistance to chemotherapy and ultimately declined standard adjuvant treatment. Another study analyzed 36 (23, 24) patients with primary hepatic neuroendocrine neoplasms who experienced recurrence after surgical resection. The results indicated that post-recurrence treatment should be individualized according to tumor characteristics and the patient’s general condition. For solitary and localized recurrent tumors, surgical treatments such as segmental or subsegmental hepatectomy were mostly adopted. For multiple tumors or patients intolerant to surgery, non-surgical treatments including chemotherapy, transcatheter arterial embolization, radiofrequency ablation, and somatostatin analogs (SSA) were selected, with significant prognostic differences among different regimens. Among them, patients who were eligible for reoperation after recurrence had relatively better overall survival, and some achieved several years of survival after re-resection. In contrast, patients receiving non-surgical treatments such as chemotherapy, TACE, radiofrequency ablation, and SSA had relatively shorter overall survival. Two recurrent patients in this study did not receive further anti-tumor therapy, mainly based on the following considerations: the patients developed severe complications such as liver failure at recurrence, with markedly abnormal liver function, manifested as significantly elevated transaminases, impaired bilirubin metabolism, and severe coagulation dysfunction. Meanwhile, the patients had poor performance status and could hardly tolerate the adverse events associated with subsequent treatment; therefore, no further anti-tumor therapy was administered. In addition, another patient in this study did not undergo surgical treatment because the tumor volume was excessively large, resulting in extremely high difficulty of surgical resection. After the pathological diagnosis was confirmed by biopsy, a multidisciplinary team (MDT) evaluation suggested that surgery and other active treatments offered limited benefits with high risks. Following thorough communication with the patient’s family about the condition and prognosis, the decision was finally made to discontinue further treatment.

Key factors affecting the prognosis of PHNEN patients include whether radical surgical treatment is possible and the tumor’s pathological grade (24, 25). Patients undergoing radical surgical resection have a better prognosis than those who do not, and NEC patients have a significantly worse prognosis than G1/G2 NET patients. One author reported (23) that the average postoperative survival time for 62 patients who underwent surgical resection was 148 months, with 1-year, 5-year, and 10-year survival rates of 98%, 80%, and 75%, respectively; whereas for 8 non-surgical patients, the average survival was 54 months, with survival rates of 75%, 33%, and 0%, respectively, concluding that the average survival time and survival rates after radical surgical resection were significantly higher than for non-surgical patients. In this study, one patient did not undergo surgery due to excessively large tumor size and survived only 3 months postoperatively, with a 0% 1-year survival rate; the 6 patients who underwent surgical resection had an average postoperative survival time of 41 months and a 100% 1-year survival rate, consistent with previous findings, indicating that radical surgical resection significantly improves patient survival rates and prolongs survival time. Furthermore, in this study, the two NEC patients recurred at 8 months and 96 months postoperatively, respectively; among the 5 G1/G2 patients, except for the one who did not undergo surgery, the remaining 4 showed no recurrence during follow-up. This suggests that the prognosis of NEC patients is significantly worse than that of G1/G2 patients. One study pointed out (26) that Ki-67 is a statistically significant predictor of tumor recurrence, with higher indices associated with a greater likelihood of postoperative recurrence. In this study, two patients recurred at 8 and 96 months postoperatively, while four postoperative patients showed no recurrence during follow-up. The median Ki-67 value in the recurrence group was 60%, markedly higher than the median Ki-67 value of 7% in the non-recurrence group. Currently, there is a lack of effective treatment for postoperative recurrence, and the survival period after recurrence is short. In this study, the two patients who recurred died within 2 months and 6 months after recurrence, respectively.

In summary, most patients with primary hepatic neuroendocrine neoplasms are discovered incidentally during physical examinations and lack obvious clinical symptoms. Plasma CgA and 24-hour urine 5-HIAA tests hold certain value for the diagnosis of PHNEN. Imaging examinations lack significant specificity. Pathological examination is the gold standard for diagnosing PHNEN, with CgA, Syn, and CD56 having the highest diagnostic value. Treatment methods for PHNEN are diverse, with radical surgical resection being the preferred approach. Whether radical surgical treatment can be performed and the tumor’s pathological grade are key factors determining patient prognosis.

## Data Availability

The original contributions presented in the study are included in the article/supplementary material. Further inquiries can be directed to the corresponding author.
